# Arthroscopic wafer procedure versus ulnar shortening osteotomy for ulnar impaction syndrome: a systematic review and meta-analysis

**DOI:** 10.1186/s13018-024-04611-4

**Published:** 2024-02-20

**Authors:** Haifeng Shi, Yongjing Huang, Yong Shen, Ke Wu, Zhihai Zhang, Qian Li

**Affiliations:** Department of Hand Surgery, Wuxi 9th People’s Hospital Affiliated to Soochow University, Wuxi, China

**Keywords:** Ulnar impaction syndrome, Arthroscopic wafer procedure, Ulnar shortening osteotomy, Meta-analysis

## Abstract

**Purpose:**

This study aimed to systematically compare the efficacy and safety of arthroscopic wafer procedure (AWP) versus ulnar shortening osteotomy (USO) for ulnar impaction syndrome (UIS) treatment.

**Methods:**

All the studies included in this meta-analysis compared the efficacy of AWP to USO for UIS and were acquired through a comprehensive search across multiple databases. The meta-analysis was performed by calculating the effect sizes with the Cochrane Collaboration’s RevMan 5.4 software.

**Results:**

A total of 8 articles were included in this analysis, comprising 148 cases in the AWP group and 163 cases in the USO group. The pooled estimates indicated no significant differences in combined Darrow’s Criteria or Modified Mayo Wrist Score, Modified Mayo Wrist Score, DASH scores, grip strength, VAS score, and postoperative ulnar variation. On the other hand, the patients in the AWP group exhibited fewer complications (OR = 0.17, 95%CI 0.05–0.54, P = 0.003) and a lower reoperation rate (OR = 0.12, 95%CI 0.05–0.28, P < 0.00001) than those in the USO group.

**Conclusions:**

The two surgical techniques were both effective in treating UIS but the AWP group showed fewer complications and a lower reoperation rate. Therefore, AWP may present a superior alternative for UIS treatment.

## Introduction

Ulnocarpal impingement syndrome (UIS) is a chronic pathology of the ulnocarpal compartment, which was first described by Friedman and Palmer as ‘‘an impingement of the ulnar head against the triangular fibrocartilage complex (TFCC) and the ulnar carpus leading to progressive degeneration of these structures’’ [1, 2]. The condition is usually associated with degenerative TFCC tears, ulnocarpal chondromalacia, lunatotriquetral ligament lesions, and positive ulnar variance. The most common symptoms include ulnar wrist pain, swelling, and limitation of motion due to excess load bearing of the ulnar head against the TFCC and ulnar carpus [3, 4].

In cases where conventional conservative treatment provides insufficient relief for UIS, surgical procedures are inevitable. Various surgical procedures have been proposed in an attempt to decrease compressive pressure across the ulnocarpal joint [5, 6]. Ulnar shortening osteotomy (USO), described by Milch et al. [7] in 1941, has been considered the gold standard for the treatment of UIS for many years. This extra-articular technique preserves the joint capsules and ligaments around the wrist joint [8, 9]. However, the procedure involves some risks of delayed union or nonunion of the ulna, hardware irritation, and secondary morphological alteration of the distal radioulnar joint (DRUJ) [10].

With the rapid development of arthroscopic technology, the arthroscopic wafer procedure (AWP), a minimally invasive procedure, has emerged as an alternative to USO for UIS treatment. It was first described by Feldon et al. as an open procedure for removing the distal 2 mm of the ulnar head beneath the horizontal section of the TFCC in 1992 [11]. Subsequently, Buterbaugh et al. [12] performed an arthroscopic version of the procedure to resect the prominent circumference of the top of the ulnar head. Despite the theoretical advantages of the AWP, the actual clinical outcomes have yet to be rigorously compared. Previous studies have compared the efficiency and safety of AWP and USO. This review aimed to provide an updated evidence synthesis comparing the two surgical procedures for UIS treatment.

## Materials and methods

This study was reported in accordance with the Preferred Reporting Items for Systematic Reviews and Meta-Analyses (PRISMA) [13] and Assessing the Methodological Quality of Systematic Reviews (AMSTAR) guidelines [14].

### Search strategy

All studies comparing the efficacy of AWP versus USO for UIS treatment, published in English or Chinese, were electronically retrieved from the Cochrane database, PubMed, Web of Science, MEDLINE, BIOSIS, Wan Fang, and CNKI EMBASE databases. Furthermore, the reference lists of the selected studies were manually searched for other eligible studies. The electronic search terms used for study retrieval were: (“ulnar impaction syndrome” OR “ulnocarpal abutment syndrome”) and (“arthroscopic wafer procedure” OR “wafer resection procedure”) and (ulnar shortening osteotomy).

### Inclusion criteria

The included articles met the following criteria: (1) patients were diagnosed with UIS; (2) all studies compared AWP versus USO for UIS; (3) randomized or non-randomized controlled clinical studies; (4) language was limited to English or Chinese; (5) a minimum sample size of 5 cases and a follow-up period of 6 months.

### Exclusion criteria

Studies were excluded if they met any of the following criteria: (1) patients were less than 16 years old; (2) additional pathological conditions of the affected extremity, including a history of trauma, combined infection, tumor, deformity, osteoporosis, or rheumatoid arthritis; (3) studies with invalid data or incomplete data; (4) duplicate studies, conference abstracts, review articles, case reports, biomechanical and cadaveric studies.

### Data extraction and management

The two authors (YJ Huang and Y Shen) screened the titles and abstracts, read the full texts according to the predetermined inclusion criteria, and independently extracted the relevant clinical information with a standardized form. The information extracted included the following: (1) author and publication year; (2) country of study; (3) study design; (4) characteristics of the patients such as age and gender; (5) sample size; (6) follow-up time; (7) the outcomes such as complications, reoperation rate, excellent and good rate, different wrist function assessments, grip strength, VAS score, and postoperative ulnar variation. Due to discrepancies in the follow-up times of the various included studies, data at the final follow-up were used for comparison.

### Risk of bias assessment

The randomized controlled studies (RCTs) were assessed based on the Cochrane Back Review Group (CBRG) [15]. If studies met at least 6 of the 11 criteria, the study was regarded as low risk of bias (RoB); otherwise, the study was labeled as high RoB. In contrast, the RoB of non-RCT studies was assessed according to the Newcastle Ottawa Quality Assessment Scale (NOQAS) [16], which has a maximum score of 9 points attributed to the quality of selection (4 points), comparability (2 points), exposure (3 points), or outcome of study participants (3 points). Scores of 0–3, 4–6, and 7–9 were regarded as high, moderate, and low RoB, respectively. The RoB of the included studies were independently assessed by two authors, and disagreements were resolved by consensus after discussion among all authors.

### Statistical analysis

The statistical analysis was performed by the RevMan 5.4 software (Cochrane IMS). To ensure an accurate analysis of the studies with missing data, the corresponding authors were contacted by email to obtain the original data. The acquired data were expressed in terms of odds ratio (OR) and 95% confidence interval (95% CI) for dichotomous variables, whereas the standardized mean difference (SMD) and 95%CI were used for continuous variables. Heterogeneity was estimated by the I^2^ statistic. A random-effects model (REM) was applied if the I^2^ value > 50% and the source of heterogeneity was measured by a subgroup analysis and/or a sensitivity analysis. Subsequently, a sensitivity analysis was performed to determine the effects of individual studies on the pooled results and evaluate the reliability of the results. Otherwise, the fixed-effects model (FEM) was applied. P < 0.05 indicated statistical significance in the integration results. Furthermore, publication bias was assessed by funnel plots following the standard methodology [17].

## Results

### Search results and characteristics of the included studies

**Fig. 1:**
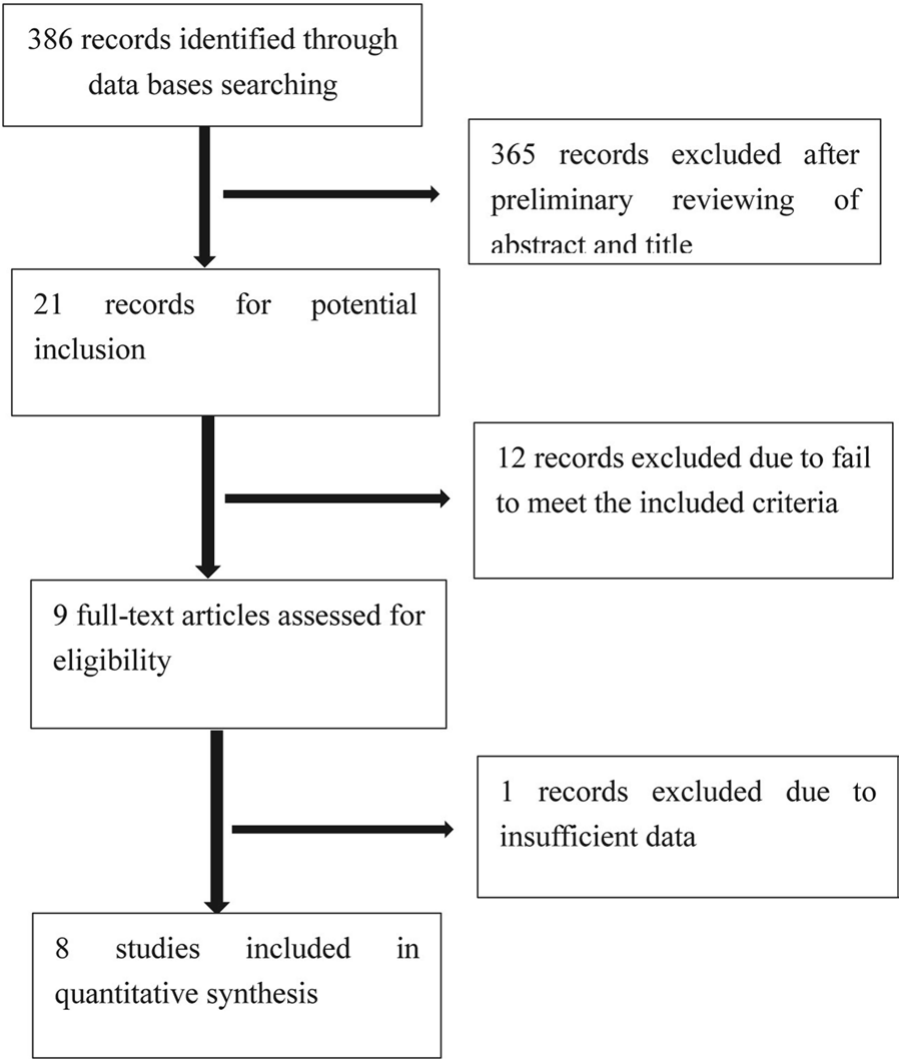
The initial search process and relevant included results

The detailed search process and relevant results are displayed in Fig. 1. A total of 8 articles [18–25], comprising 2 RCTs [22, 25] and 6 retrospective studies [18–21, 23, 24], were included in the analysis. Overall, the studies involved 148 patients in the AWP group and 163 patients in the USO group. The participants of three studies were Mongoloids and others were Caucasians. The characteristics of the included studies are summarized in Table 1.

**Table 1:** The characteristics of the included studies

Study ID	Study design	Country	Case		Sex (M/F*)		Age (year)		Affected hand (L/R*)		Follow-up (months)	
			AWP	USO	AWP	USO	AWP	USO	AWP	USO	AWP	USO
Bernstein 2004	RCS	USA	11	16	6/5	8/8	24–61	19–65	UA*	UA	7–61	7–58
Constantine 2000	RCS	USA	11	11	5/6	3/8	46	35	UA	UA	26	46
Auzias 2020	RCS	France	24	9	UA	UA	44.4 ± 12	39.1 ± 4	UA	UA	55 + − 4	103 ± 8
Oh 2018	RCS	Korea	19	23	8/11	9/14	53.8 ± 7.0	53.5 ± 6.6	14/5	10/13	34.6 ± 12.0	36.2 ± 11.6
Teng 2020	RCT	China	19	23	13/6	16/7	UA	UA	7/12	8/15	14.61 ± 2.19	14.61 ± 2.19
Chen 2020	RCS	China	22	23	13/9	13/10	38.4 ± 9.5	37.3 ± 7.1	6/16	6/17	13.7	14.3
Smet 2014	RCS	Belgium	12	28	8/4	22/6	31–66	16–61	UA	UA	UA	UA
Afifi 2022	RCT	Egypt	30	30	18/12	26/4	UA	UA	4/26	10/20	22 ± 5.7	21.1 ± 5.3

AWP * means arthroscopic wafer procedure

USO* means ulnar shortening osteotomy

M/F* means male/female

L/R* means left/right

UA* means data are unavailable

RCT* means Randomized Controlled Trials

RCS* means Retrospective Cohort Study

### Study quality assessment

The methodological quality of the two RCTs showed a low RoB, with scores of 7 and 9. Among the non-RCTs, 3 studies had a low RoB, while the remaining 3 studies had moderate RoB. Overall, the risk of bias in this study was low to moderate. The quality assessment of non-RCTs is displayed in Table 2.

**Table 2:** Quality assessment according to the Newcastle–Ottawa scale of the non-randomized studies

Study ID	Selection	Comparability	Exposure	Total score
Bernstein 2004	2	1	2	5
Constantine 2000	2	1	1	4
Auzias 2020	4	1	2	7
Oh 2018	3	2	2	7
Chen 2020	3	2	2	7
Smet 2014	2	1	1	4

### Meta-analysis results

#### Complications

**Fig. 2:**
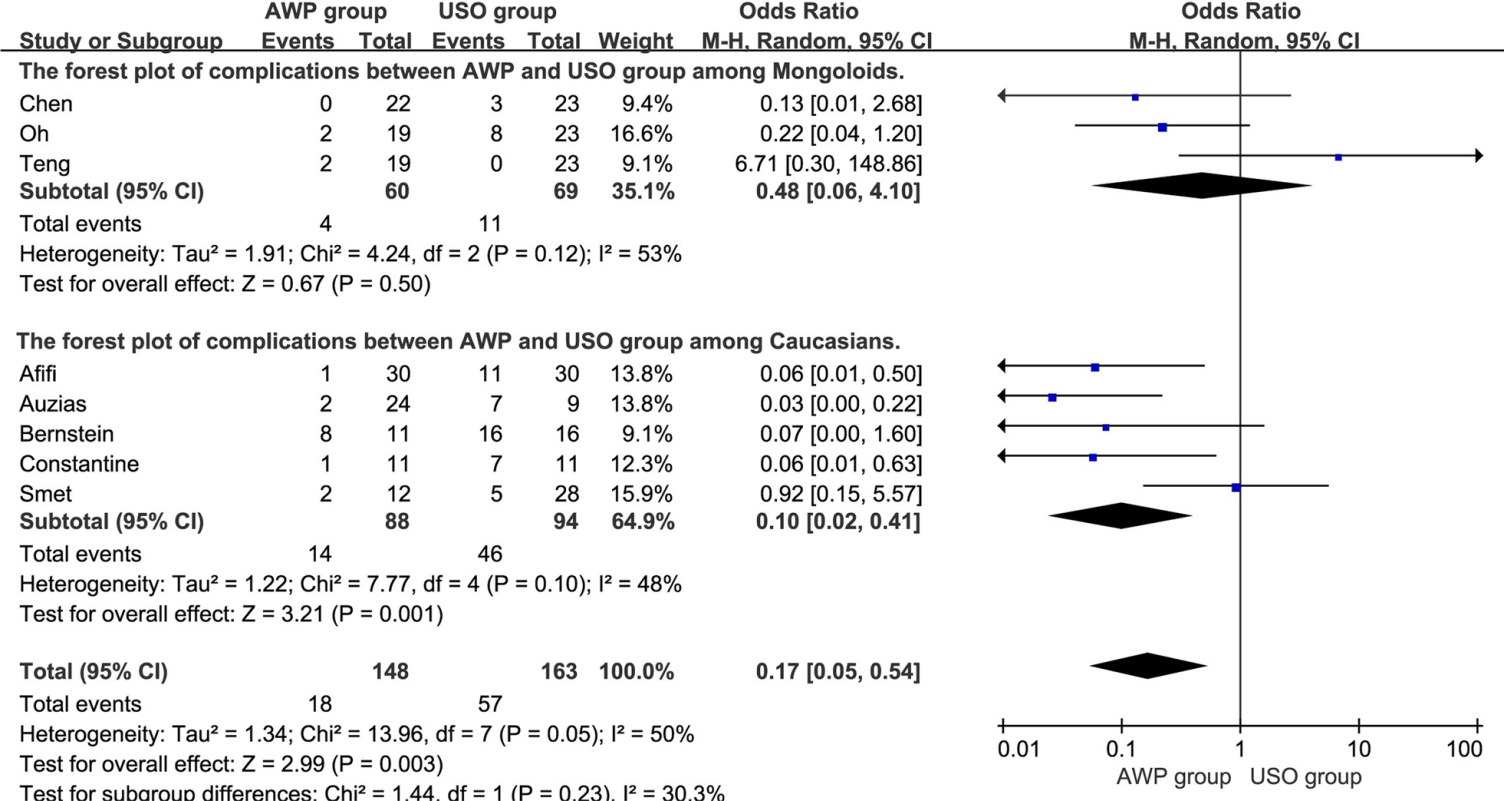
The forest plot of complications between AWP and USO group and subgroup analysis. **2.1.1** The forest plot of complications between AWP and USO group among Mongoloids. **2.1.2** The forest plot of complications between AWP and USO group among Caucasians

All 8 articles evaluated the complications associated with the surgical procedures. Significant heterogeneity was observed (I^2^ = 50%; P = 0.05) and a REM was applied. The results of the pooled analysis revealed a significant difference in complications between the two groups (OR = 0.17, 95%CI, 0.05–0.54, P = 0.003). Subsequently, a subgroup analysis showed significant differences between the two groups among Caucasians (OR = 0.10, 95%CI, 0.02–0.41, P = 0.001) but not among Mongoloids (OR = 0.48, 95%CI, 0.06–4.10, P = 0.50) (Fig. 2).

### Reoperation rate

**Fig. 3:**
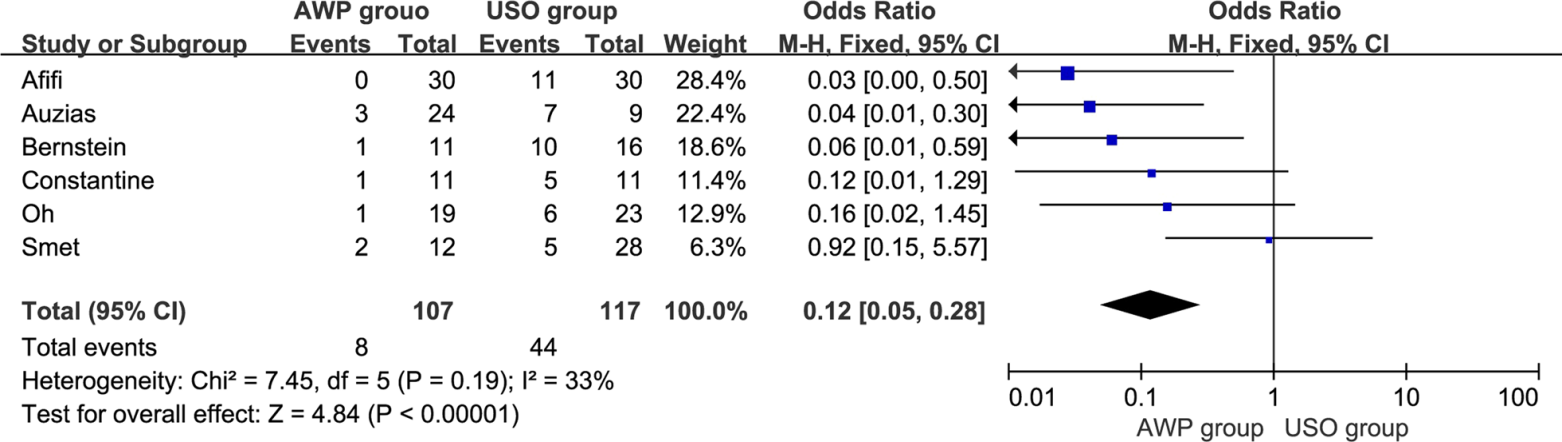
The forest plot of reoperation rate between AWP and USO group

A total of 6 studies [18–21, 24, 25], including 107 patients in the AWP group and 117 patients in the USO group, reported the reoperation rate. The pooled results showed no significant heterogeneity from these trials (I^2^ = 33%, P = 0.19), and a FEM was applied. The statistical results indicated a significant difference between the two surgical groups (OR = 0.12, 95%CI, 0.05–0.28, P < 0.00001) (Fig. 3).

### Excellent and good rate

**Fig. 4:**
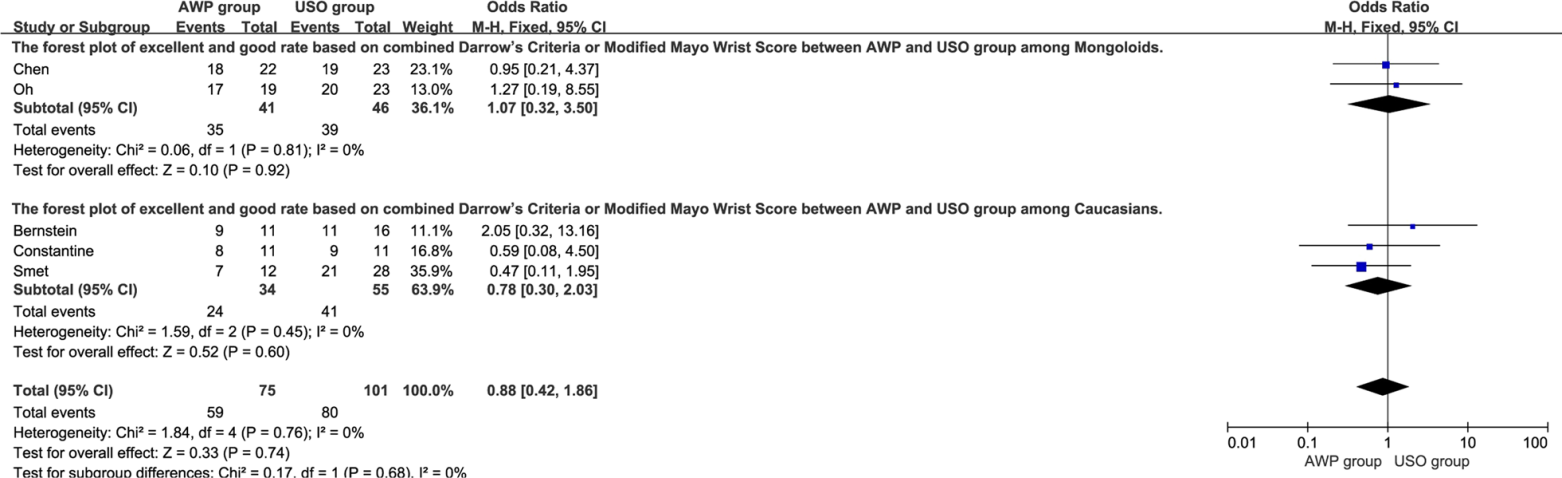
The forest plot of excellent and good rate based on combined Darrow’s Criteria or Modified Mayo Wrist Score between AWP and USO group and subgroup analysis. **4.1.1** The forest plot of excellent and good rate based on combined Darrow’s Criteria or Modified Mayo Wrist Score between AWP and USO group among Mongoloids. **4.1.2** The forest plot of excellent and good rate based on combined Darrow’s Criteria or Modified Mayo Wrist Score between AWP and USO group among Caucasians

**Fig. 5:**
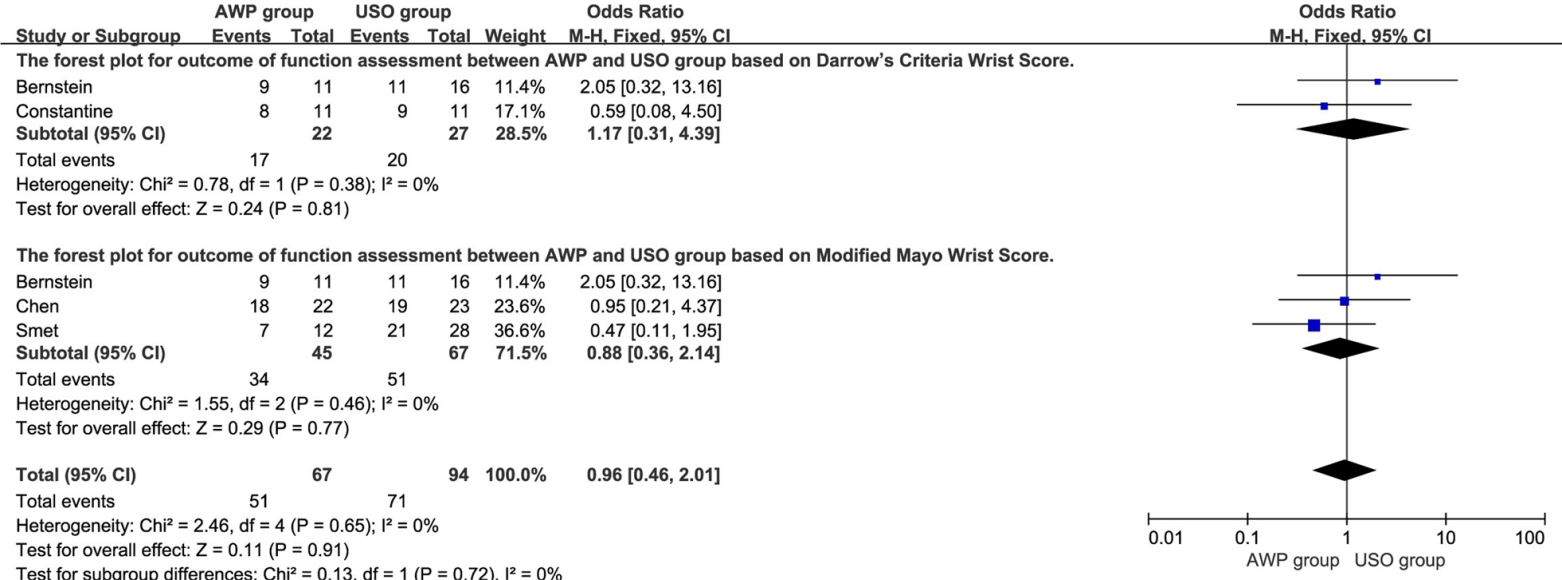
The forest plot for excellent and good rate based on different function assessments between AWP and USO group and subgroup analysis. **5.1.1** The forest plot for excellent and good rate between AWP and USO group based on Darrow’s Criteria Wrist Score. **5.1.2** The forest plot for excellent and good rate between AWP and USO group based on Modified Mayo Wrist Score

Data related to excellent and good rate based on different wrist function assessment forms at the final follow-up was reported in 5 trials [18, 19, 21, 23, 24]. Firstly, we compared the excellent and good rate based on combined Darrow’s Criteria or Modified Mayo Wrist Score. The overall pooled results showed no significant difference between the two groups (OR = 0.88, 95%CI, 0.42–1.86, P = 0.74), which was consistent with the subgroup analysis among Caucasians (OR = 0.78, 95%CI, 0.30–2.03, P = 0.60) and Mongoloids (OR = 1.07, 95%CI, 0.32–3.50, P = 0.92) (Fig. 4). Moreover, the excellent and good rates were compared based on the individual Darrow’s Criteria and Modified Mayo Wrist Score. No significant difference (OR = 0.96, 95%CI, 0.46–2.01, P = 0.91) was found between the two groups as well as the subgroup analysis (Fig. 5).

### Modified mayo wrist score

**Fig. 6:**
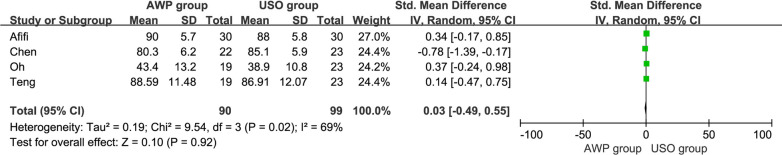
The forest plot of the Modified Mayo Wrist Score between AWP and USO group

A total of 4 studies [21–23, 25], including 90 cases in the AWP group and 99 cases in the USO group, reported the Modified Mayo Wrist Score. Significant heterogeneity (I^2^ = 69%; P = 0.02) was detected among the trials. A REM was established and the pooled results showed no significant difference between the AWP and the USO group (SMD = 0.03, 95%CI, − 0.49 to 0.55, P = 0.92) (Fig. 6). A sensitivity analysis was performed, demonstrating that no study significantly influenced the results.

### DASH scores

**Fig. 7:**
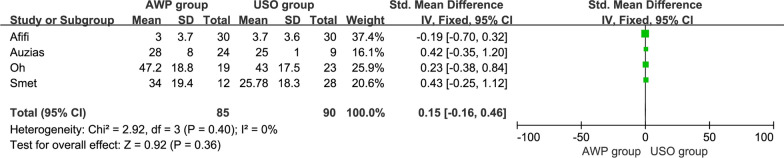
The forest plot of the DASH scores between AWP and USO group

DASH scores were reported in 4 articles [20, 21, 24, 25], which included 85 participants in the AWP group and 90 participants in the USO group. The statistical results suggested no significant heterogeneity (I^2^ = 0%, P = 0.40), and no significant difference was observed between the two procedures (SMD = 0.15, 95% CI, − 0.16 to 0.46) (Fig. 7).

### Grip strength

**Fig. 8:**
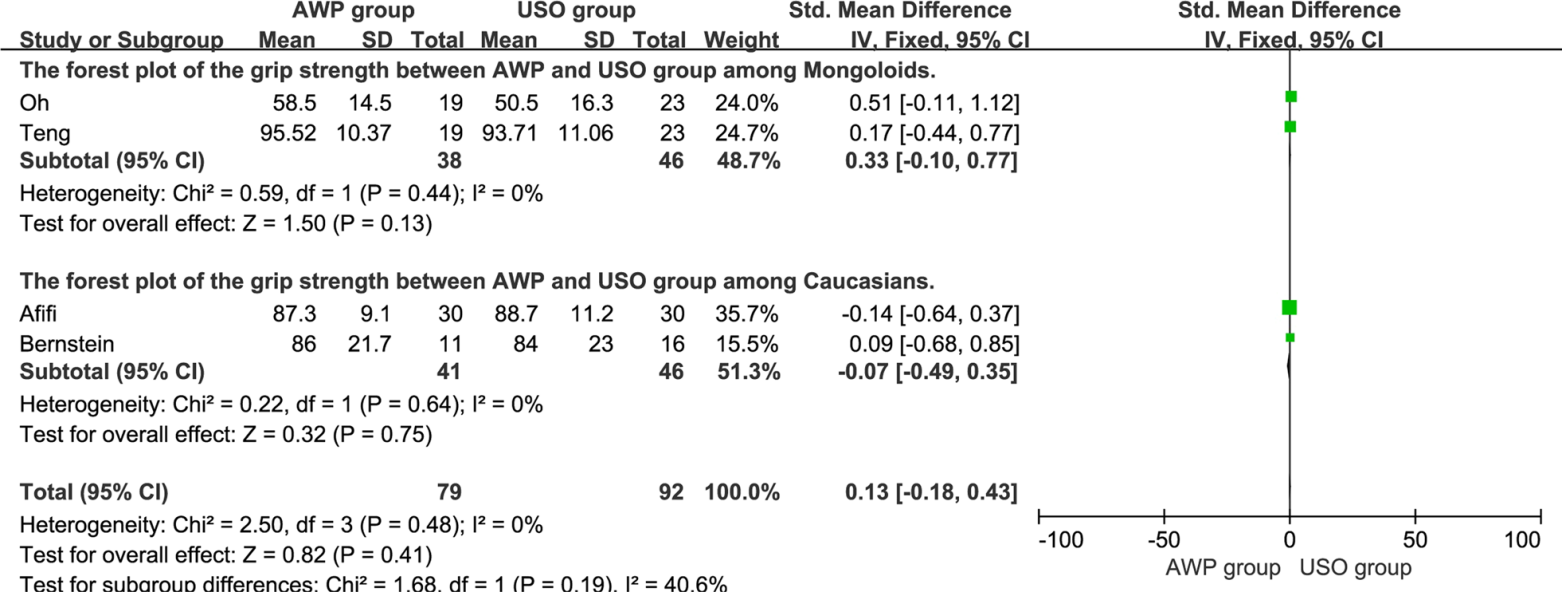
The forest plot of the grip strength between AWP and USO group and subgroup analysis. **8.1.1** The forest plot of the grip strength between AWP and USO group among Mongoloids. **8.1.2** The forest plot of the grip strength between AWP and USO group among Caucasians

Grip strength was described in 4 studies [18, 21, 22, 25], including 79 cases in the AWP group and 92 cases in the USO group. The overall pooled results showed no significant difference between the two groups (SMD = 0.13, 95%CI, − 0.18 to 0.43, P = 0.41), and the subgroup analysis confirmed the same results among Caucasians and Mongoloids (Fig. 8).

### VAS score

**Fig. 9:**
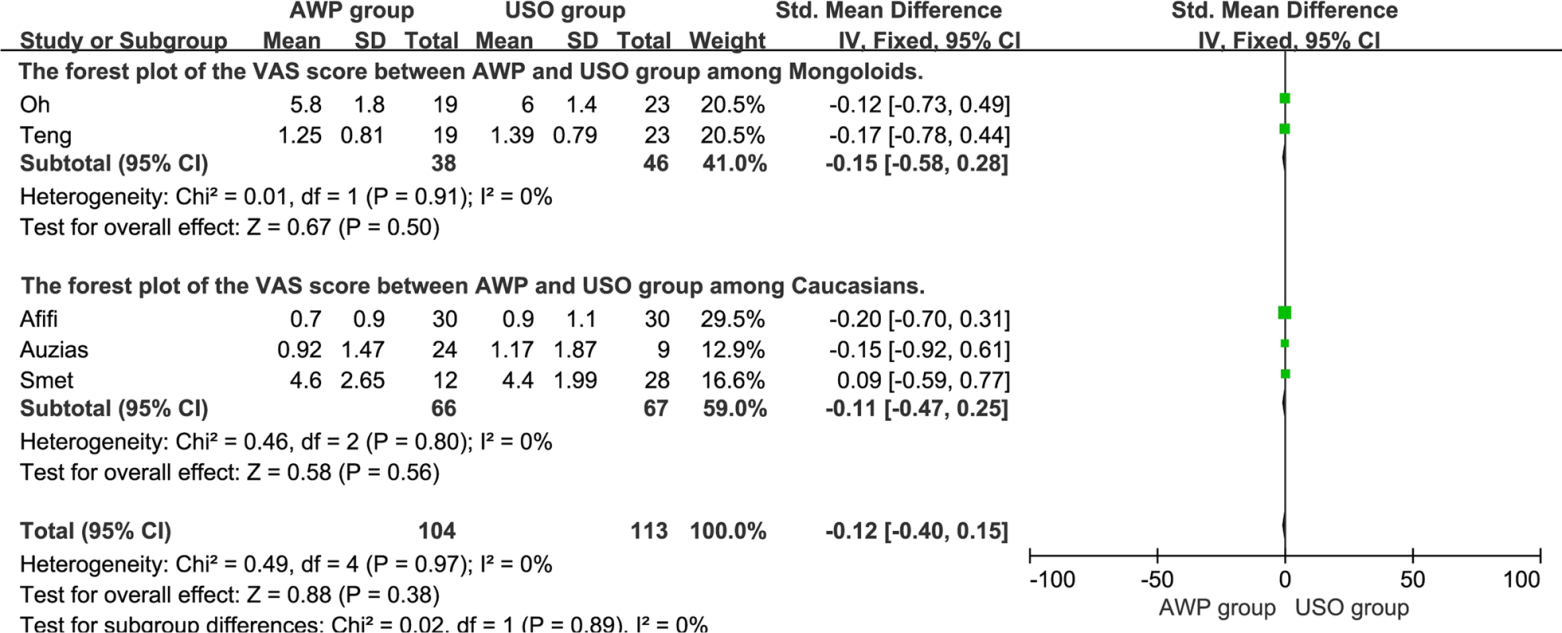
The forest plot of the VAS score between AWP and USO group and subgroup analysis. **9.2.1** The forest plot of the VAS score between AWP and USO group among Mongoloids. **9.2.2** The forest plot of the VAS score between AWP and USO group among Caucasians

A total of 5 articles [18, 21, 22, 24, 25] reported the VAS score at the final follow-up, with 104 patients in the AWP group and 113 patients in the USO group. The overall pooled results showed no significant difference between the two groups (SMD = − 0.12, 95%CI, − 0.40 to 0.15, P = 0.38), with the subgroup analysis showing similar results among Caucasians and Mongoloids (Fig. 9).

### Postoperative ulnar variation

**Fig. 10:**
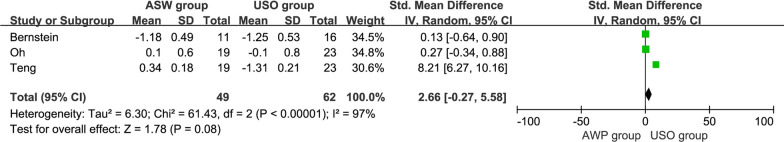
The forest plot of the postoperative ulnar variation between AWP and USO group

Useful data on postoperative ulnar variation were only available in 3 studies [18, 21, 22], including 49 participants in the AWP group and 62 participants in the USO group. The results showed no significant difference between the two groups (SMD = 2.66, 95%CI, − 0.27 to 5.58, P = 0.08) (Fig. 10). Sensitivity analysis was performed and no study was found to significantly influence the results.

## Discussion

Owing to ulnar positive variance affecting load transfer across the wrist in UIS, surgical procedures that shorten the ulna may provide theoretical improvement [26, 27]. USO has been applied for a variety of ulnar-sided wrist disorders. Ulnar shortening provides mechanical decompression of the ulnocarpal joint by transferring the load to the radiocarpal joint, thereby improving UIS [6, 28]. Palmer et al. [29] found that 18% of the loading pressure across the wrist was borne by the ulnocarpal articulation, which could be reduced to 4.3% by shortening the ulna by 2.5 mm. The main advantages of this procedure include maintaining the integrity of the DRUJ, its surrounding ligaments, and the joint capsule [30, 31]. Meanwhile, some modified methods have used oblique osteotomy and osseous compression techniques with or without the use of special compression devices [32, 33] or commercial plates designed specifically for this procedure [34]. Some studies have reported that this procedure could relieve pain, and improve grip strength and wrist functional scores [35–37]. However, the procedure is plagued by multiple complications [38]. In addition, USO is not suitable for patients with a reverse oblique inclination of the DRUJ due to the resultant joint incongruity [39, 40].

The arthroscopic wafer procedure is an effective technique to decompress ulnar-positive wrists, along with debriding TFCC tears, which circumvents the risks of osteotomy, hardware complications, and bony union [41]. However, the reduction in ulnocarpal transmission forces in AWP is limited by its relatively low ulnar shortening capacity (not exceeding 4 mm) [42, 43]. Further shortening may reduce the contact of the DRUJ articular surfaces and increase DRUJ pressure, which may cause osteoarthritis. Furthermore, the complications of wrist arthroscopy mainly include portal site infection, neurapraxia of the dorsal cutaneous branch of the ulnar nerve, and wrist stiffness [44, 45]. Neither surgical procedure has demonstrated superiority over the other, but some studies have compared the two surgical techniques for UIS. Consequently, this meta-analysis was performed to synthesize the currently available evidence to examine the efficacy and safety of the two procedures.

Based on the above results, the two surgical techniques were found to yield equivalent improvements in pain relief, grip strength, and postoperative wrist function assessments. Teng et al. [22] and Afifi et al. [25] reported that the two procedures achieved comparable pain relief, grip strength, and wrist functional improvement at the final follow-up in their RCTs. Likewise, similar conclusions were achieved in other retrospective studies included in this meta-analysis. However, patients in the AWP group exhibited fewer complications than those in the USO group. Interestingly, similar results were observed among Caucasians but not among Mongoloids. Hardware irritation and bony non-union have traditionally been regarded as being the primary cause of complications, but continuous improvement of surgical techniques and internal fixation materials for USO have decreased their impact. The studies involving Mongoloids were performed later than those on Caucasians, which may be an important factor for the above results. Furthermore, lower reoperation rates were observed in AWP compared to USO, which is consistent with most previous studies. Fixation-related issues are the main reasons for secondary operations for USO. Chan et al. [46] reported an incidence of plate removal for symptomatic hardware irritation as high as 45% in 63 consecutive cases. In addition, Benis et al. [47] reported a 50% rate of revision surgery for hardware removal. Nonunion or delayed union are other common causes of reoperation following the USO technique. Owens et al. [48] described an average rate of nonunion among all osteotomies of 4.0% in a systematic review. In this study, the incidence of both nonunion and delayed union rates was 2.45%. Refracture of the ulna after hardware removal, complaints of DRUJ, and other complications have shown a lower incidence. AWP avoids the above complications but leads to DRUJ stability and an increased risk of osteoarthritis. It is a degenerative process that is easily overlooked [42, 49]. Ulnar variance is an important factor following UIS surgery, and most authors suggest achieving a postoperative ulnar variance of 0 to − 1 mm [50, 51]. The pooled results indicated that the two surgical techniques could achieve similar postoperative ulnar variance.

For UIS with advanced DRUJ arthritis, surgical treatments aim to eliminate the articulation between the distal ulna and radius by resecting all or a portion of the distal ulna, fusing the joint, or replacing the distal ulna. Surgical options include the Darrach procedure, the Sauvé-Kapandji operation, the hemiresection–interposition technique, implant arthroplasty, and wide distal ulnar resection. Each of these techniques has advantages and disadvantages that should be considered when planning a procedure [52].

Nevertheless, the limitation of the present analysis should be acknowledged. First, most included articles were retrospective studies and the absence of high-quality evidence regarding interventions may introduce selection bias. In addition, the sample size of comparative studies was small. Moreover, clinical heterogeneity such as age, occupation of patients, or surgeon skill proficiency and experience were inevitable. Finally, the follow-up times varied among the studies and may have potentially influenced the results.

## Conclusion

The two surgical procedures were both effective in improving pain, grip strength, and wrist function. However, for appropriate patients of UIS, AWP may be a better alternative due to the lower rate of complications and reoperation compared with USO. Further high-quality studies with large sample sizes are required to provide more robust evidence on this topic.

## Data Availability

The datasets used and/or analyzed during the current study are available from the corresponding author on reasonable request.

## References

[1] Chun S, Palmer AK (1993). The ulnar impaction syndrome: follow-up of ulnar shortening osteotomy. J Hand Surg Am.

[2] Tomaino MM. Ulnar impaction syndrome in the ulnar negative and neutral wrist. Diagnosis and pathoanatomy. J Hand Surg Br 1998; 23:754–7. 10.1016/s0266-7681(98)80090-9 9888675

[3] Sammer DM, Rizzo M (2010). Ulnar impaction. Hand Clin.

[4] Katz DI, Seiler JG, Bond TC (2010). The treatment of ulnar impaction syndrome: a systematic review of the literature. J Surg Orthop Adv..

[5] Stockton DJ, Pelletier ME, Pike JM (2015). Operative treatment of ulnar impaction syndrome: a systematic review. J Hand Surg Eur.

[6] Minami A, Kato H (1998). Ulnar shortening for triangular fibrocartilage complex tears associated with ulnar positive variance. J Hand Surg Am.

[7] Milch H (1942). So-called dislocation of the lower end of the ulna. Ann Surg.

[8] Clark SM, Geissler WB (2012). Results of ulnar shortening osteotomy with a new plate compression system. Hand (NY).

[9] Sraj SA, Budoff JE (2009). Ulnar or radial shortening osteotomy with a single saw cut. J Hand Surg Am..

[10] Krimmer H, Unglaub F, Langer MF (2016). The distal radial decompression osteotomy for ulnar impingement syndrome. Arch Orthop Trauma Surg..

[11] Feldon P, Terrono AL, Belsky MR (1992). The ‘‘wafer’’ procedure. Partial distal ulnar resection. Clin Orthop Relat Res..

[12] Buterbaugh GA (1992). Ulnar impaction syndrome: treatment by arthroscopic removal of the distal ulna. Tech Orthop.

[13] Page MJ, McKenzie JE, Bossuyt PM (2021). The PRISMA 2020 statement: an updated guideline for reporting systematic reviews. BMJ.

[14] Shea BJ, Reeves BC, Wells G (2017). AMSTAR 2: a critical appraisal tool for systematic reviews that include randomised or non-randomised studies of healthcare interventions, or both. BMJ.

[15] Furlan AD, Pennick V, Bombardier C (2009). Cochrane Back Review Group 2009 updated method guidelines for systematic reviews in the Cochrane Back Review Group. Spine (Phila Pa 1976).

[16] Stang A (2010). Critical evaluation of the Newcastle-Ottawa scale for the assessment of the quality of nonrandomized studies in meta-analyses. Eur J Epidemiol.

[17] Higgins JP, Thompson SG (2002). Quantifying heterogeneity in a meta-analysis. Stat Med.

[18] Bernstein MA, Nagle DJ, Martinez A (2004). A comparison of combined arthroscopic triangular fibrocartilage complex debridement and arthroscopic wafer distal ulna resection versus arthroscopic triangular fibrocartilage complex debridement and ulnar shortening osteotomy for ulnocarpal abutment syndrome. Arthroscopy.

[19] Constantine KJ, Tomaino MM, Herndon JH (2000). Comparison of ulnar shortening osteotomy and the wafer resection procedure as treatment for ulnar impaction syndrome. J Hand Surg Am.

[20] Auzias P, Delarue R, Camus EJ (2021). Ulna shortening osteotomy versus arthroscopic wafer procedure in the treatment of ulnocarpal impingement syndrome. Hand Surg Rehabil.

[21] Oh WT, Kang HJ, Chun YM (2018). Arthroscopic wafer procedure versus ulnar shortening osteotomy as a surgical treatment for idiopathic ulnar impaction syndrome. Arthroscopy.

[22] Teng J, Li G, Wang M (2020). Arthroscopic Wafer procedure versus open ulnar shortening osteotomy for ulnar impingement syndrome. Orthop J China.

[23] Chen H, Teng X, Yuan H (2020). Wrist arthroscopy-assisted ulnar head wafer resection versus ulnar shortening osteotomy for treatment of ulnar impaction syndrome. Chin J Orthop Trauma.

[24] Smet LD, Vandenberghe L, Degreef I (2014). Ulnar impaction syndrome: ulnar shortening vs. Arthroscopic wafer procedure. J Wrist Surg.

[25] Afifi A, Ali AM, Abdelaziz A (2022). Arthroscopic wafer procedure versus ulnar shortening osteotomy for treatment of idiopathic ulnar impaction syndrome: a randomized controlled trial. J Hand Surg Am.

[26] Loh YC, Van Den Abbele K (1999). The results of ulnar shortening for ulnar impaction syndrome. J Hand Surg.

[27] Iwasaki N, Ishikawa J, Kato H (2007). Factors affecting results of ulnar shortening for ulnar impaction syndrome. Clin Orthop Relat Res.

[28] Trumble TE, Gilbert M, Vedder N (1997). Ulnar shortening combined with arthroscopic repairs in the delayed management of triangular fibrocartilage complex tears. J Hand Surg Am.

[29] Werner FW, Glisson RR, Murphy DJ (1986). Force transmission through the distal radioulnar carpal joint: effect of ulnar lengthening and shortening. Handchir Mikrochir Plast Chir.

[30] Feldon P, Terrono AL, Belsky MR (1992). Wafer distal ulna resection for triangular fibrocartilage tears and/or ulna impaction syndrome. J Hand Surg Am.

[31] Thiru RG, Ferlic DC, Clayton ML (1986). Arterial anatomy of the triangular fibrocartilage of the wrist and its surgical significance. J Hand Surg Am.

[32] Chen NC, Wolfe SW (2003). Ulna shortening osteotomy using a compression device. J Hand Surg [Am].

[33] Wehbe MA, Cautilli DA (1995). Ulnar shortening using the AO small distractor. J Hand Surg [Am].

[34] Rayhack JM, Gasser SI (1993). Precision oblique osteotomy for shortening of the ulna. J Hand Surg [Am].

[35] Acott TR, Greenberg JA (2020). Ulnar abutment syndrome in the athlete. Orthop Clin North Am..

[36] Rayhack JM, Gasser SI, Latta LL (1993). Precision oblique osteotomy for shortening of the ulna. J Hand Surg Am.

[37] McBeath R, Katolik LI, Shin EK (2013). Ulnar shortening osteotomy for ulnar impaction syndrome. J Hand Surg Am.

[38] Cardoso ANP, Viegas R, Gamelas P (2020). Ulnar shortening osteotomy: our experience. Rev Bras Ortop (Sao Paulo).

[39] Tomaino MM (2018). Editorial commentary: wrist ulnar impaction syndrome: when I use the wafer procedure and when I do not. Arthroscopy.

[40] Sagerman SD, Zogby RG, Palmer AK (1995). Relative articular inclination of the distal radioulnar joint: a radiographic study. J Hand Surg Am..

[41] Loh YC, Van Den Abbeele K, Stanley JK (1999). The results of ulnar shortening for ulnar impaction syndrome. J Hand Surg Br.

[42] Colantoni JG, Chadderdon C, Gaston RG (2014). Arthroscopic wafer procedure for ulnar impaction syndrome. Arthrosc Tech..

[43] Markolf KL, Tejwani SG, Benhaim P (2005). Effects of wafer resection and hemiresection from distal ulna on load-sharing at the wrist: a cadaveric study. J Hand Surg Am.

[44] Wnorowski DC, Palmer AK, Werner FW (1992). Anatomic and biomechanical analysis of the arthroscopic wafer procedure. Arthroscopy.

[45] Griska A, Feldon P (2015). Wafer resection of the distal ulna. J Hand Surg Am.

[46] Chan SK, Singh T, Pinder R (2015). Ulnar shortening osteotomy: are complications under reported?. J Hand Microsurg.

[47] Benis S, Goubau JF, Mermuys K (2017). The oblique metaphyseal shortening osteotomy of the distal ulna: surgical technique and results of ten patients. J Wrist Surg.

[48] Owens J, Compton J, Day M (2019). Nonunion rates among ulnarshortening osteotomy for ulnar impaction syndrome: a systematic review. J Hand Surg Am.

[49] Jang E, Danoff JR, Rajfer RA (2014). Revision wrist arthroscopy after failed primary arthroscopic treatment. J Wrist Surg.

[50] Darlis NA, Ferraz IC, Kaufmann RW (2005). Step-cut distal ulnar-shortening osteotomy. J Hand Surg Am.

[51] Tomaino MM, Weiser RW (2001). Combined arthroscopic TFCC debridement and wafer resection of the distal ulna in wrists with triangular fibrocartilage complex tears and positive ulnar variance. J Hand Surg Am.

[52] Faucher GK, Zimmerman RM, Zimmerman NB (2016). Instability and arthritis of the distal radioulnar joint: a critical analysis review. JBJS Rev.

